# Awareness of Breast Cancer among Female Students and Faculty from Najran University, Najran, Saudi Arabia

**DOI:** 10.31557/APJCP.2020.21.5.1415

**Published:** 2020-05

**Authors:** Saeed Ali Alsareii, Saad Mansour Alqahtani, Abdulrahman Manaa Alamri, Hajr Hassan Al-Wadei, Samer Ali Al-ammari, Awad Mohammed Al-Qahtani, Ahmed Abu-Zaid

**Affiliations:** 1 *Department of Surgery, College of Medicine, Najran University, Najran, Saudi Arabia. *; 2 *Department of Surgery, College of Medicine, Majmaah University, Majmaah, Saudi Arabia. *; 3 *Department of Surgery, King Khalid Hospital, Najran, Saudi Arabia. *; 4 *Department of Family and Community Medicine, College of Medicine, Najran University, Najran, Saudi Arabia. *; 5 *College of Medicine, Alfaisal University, Riyadh, Saudi Arabia. *; 6 *College of Graduate Health Sciences, University of Tennessee Health Science Center, Memphis, Tennessee, United States. *

**Keywords:** Awareness, breast cancer, Najran, Saudi Arabia

## Abstract

**Background:**

Breast cancer (BC) is the leading malignancy among women in Najran, Saudi Arabia. However, not much is known about the public’s awareness of BC. This study explored the general knowledge, early warning signs, risk factors and sources of information about BC.

**Methods:**

An online-based, anonymous, self-rating, cross-sectional and survey-based study was conducted from March-2019 to April-2019. Three-hundred female students and/or faculty from College of Medicine, Najran University (Najran, Saudi Arabia) participated in the study.

**Results:**

A total of 232 students (77.3%) and 68 faculty (22.7%) responded to the survey. Our study showed that nulliparity (83.8%) and early menarche before 12 years of age (29.7%) were the most pertinent obstetric risk factors of BC. Conversely, lack of physical activity (66.3%) and family history of BC (18%) were the most substantial non-obstetric risk factors of BC. According to pre-defined criteria, while the surveyed research subjects demonstrated ‘good’ general knowledge about BC (75.3%), they unfavorably exhibited ‘poor’ knowledge about the warning signs of BC (94.3%). The predictors of ‘good’ overall knowledge (general knowledge plus signs knowledge about BC) included age, marital status, educational level and family history (all p<0.05, two-tailed Chi-square test). Apart from the campaigns’ educational materials (43%), the top source of knowledge about BC was internet (33%), whereas the lowest ones were healthcare professionals (11.3%) and training workshops (7.3%).

**Conclusions:**

The surveyed research subjects harbored risk factors of BC and demonstrated ‘poor’ knowledge about the warning early signs of BC. We call for rigorous and well-crafted educational campaigns geared toward improving the awareness level of BC among women in Najran province.

## Introduction

In Saudi Arabia, breast cancer (BC) is the most common malignancy in women, as well as the leading cause of cancer-related mortality (The Global Cancer Observatory, 2018). A recently published report pinpointed that the number of BC-related deaths is anticipated to increase by two folds between 2025 and 2050 (Alattas, 2019). The clinical stage of BC at the time of presentation is regarded as one of the most substantial prognostic factors of survival (Siegel et al., 2019). The five-year overall survival rates for patients with localized and regional diseases are very favorable, and may reach up to 99% and 85%, respectively (Siegel et al., 2019). Conversely, the five-year overall survival rate for patients with metastatic disease is very dismal, and may drop down significantly to as low as 27% (Siegel et al., 2019). Alotaibi et al., (2018) reviewed the BC mortality in Saudi Arabia over a nine-year period from 2004 to 2013; they demonstrated that nearly 45% of BC-related deaths occurred in patients with advanced-stage disease as opposed to 12% in patients with early-stage disease. Unfortunately, in Saudi Arabia, only a small proportion of Saudi BC women (approximately 27-38%) presents to clinical attention with early-stage disease (Ministry of Health in Saudi Arabia, 2012; Nageeti et al., 2017; National Health Information Center for the Saudi Cancer Registry, 2019).

Generally, the risk factors of BC are dichotomized into modifiable and non-modifiable risk factors (CDC, 2018). Examples of modifiable risk factors include weight, diet, exercise, smoking, alcohol consumption, use of oral contraceptive pills, stress and anxiety (CDC, 2018). On the other hand, examples of non-modifiable risk factors include genetic predisposition, gender, age, family history, age of menarche, breastfeeding, age of menopause and radiation exposure to the chest (CDC, 2018). These factors, individually or in an amalgamation, can trigger the pathogenesis of BC. Adequate awareness of BC-related risk factors and early warning signs is instrumental in reducing the BC-associated morbidity and mortality burdens. 

Prevention, early detection and prompt treatment of BC are key elements in improving survival and the quality of life in patients with BC (Alshahrani et al., 2019a). Breast self-examination (BSE), clinical breast examination (CBE) and mammogram are the most common means of BC screening, and have been shown to markedly lessen the morbidity and mortality of BC by promoting timely detection and swift treatment (Tabar et al., 1999; Coleman, 2017). Thus, ample awareness and facilitation of such screening methods is crucially important.

A number of studies in Saudi Arabia endeavored to scrutinize the public’s awareness of BC, in terms of its early signs/symptoms, risk factors and screening methods (Amin et al., 2009; Radi, 2013; Alrashidi et al., 2017; Al Otaibi et al., 2017; Nageeti et al., 2017; Al-Zalabani et al., 2018; Binhussien and Ghoraba, 2018; Hussein et al., 2018; Alshahrani et al., 2019a; Alshahrani et al., 2019b). Overall, these studies demonstrated an unfavorable low awareness level of BC. The vast majority of these studies originated from the central, northern, eastern and western regions of Saudi Arabia. For each city examined within the former mentioned regions, there are at least two or more reported studies about BC awareness. However, with respect to the southern region of Saudi Arabia, a very limited figure of studies has been reported to date (Mahfouz et al., 2013; Alshahrani et al., 2019a; Alshahrani et al., 2019b; Madkhali et al., 2019). The southern region of Saudi Arabia includes Asir, Baha, Jazan and Najran, and has been shown to harbor the lowest incidence rate of BC (National Health Information Center for the Saudi Cancer Registry, 2019).

Najran, located in the southern region of Saudi Arabia, has an estimated population of around 510,000 individuals. According to the most recent Cancer Incidence Report (2015) published by the Saudi Cancer Registry, BC is the leading malignancy overall including both genders (14.4%) as well as among women specifically (24.7%) (National Health Information Center for the Saudi Cancer Registry, 2019). To the best of knowledge, only one study has been recorded so far from Najran about the awareness level of BC among women (Alshahrani et al., 2019a). The study surveyed 500 females who attended primary healthcare centers, however, it did not explore the inherent risk factors and signs knowledge of BC among the surveyed research subjects. We, the authors, believe that more studies are needed about the awareness level of BC in Najran—a region that is underrepresented. Such studies are critically important to supplement literature with additional data so that accordingly strengths can be reinforced, whereas weaknesses can be rectified. 

Our study has four specific aims: (i) to explore the obstetric and non-obstetric risk factors of BC, (ii) to examine the general and specific signs knowledge domains of BC, (iii) to scrutinize the determinants of overall knowledge of BC, and (iv) to investigate the sources of knowledge of BC among female students and faculty from Najran University, Najran, Saudi Arabia. 

## Materials and Methods

*Study design, setting and ethical approval*

An online-based, anonymous, self-rating, cross-sectional and survey-based study was conducted from March 2019 to April 2019 at Najran University, Najran, Saudi Arabia. The study research subjects included only female students and faculty, which is a cohort of research subjects that has not been surveyed before by an earlier study conducted in Najran. Participation in the study was voluntary and no compensation was provided. By agreeing to take part in the study, the research participants provided an implied consent. Taking into account a 95% confidence interval and 5% margin of error, the study sample size was computed to be a minimum of 287 female individuals (students and/or faculty). In order to account for unforeseen circumstances, a target of 300 research participants was determined. The study protocol was approved by the Institutional Review Board (IRB) and Ethics Committee at Najran University, Najran, Saudi Arabia.

*Study survey*

According to a literature search and scrutiny of previous studies (Amin et al., 2009; Alshahrani et al., 2019a; Al-Zalabani et al., 2019), the survey questions were constructed by three authors (SAA, AMA and HHA). Afterwards, the survey was reviewed by the remaining authors for content, accuracy and lucidity of questions. Then, the survey was piloted on a group of 10 female students to double-check for face validity and apt understanding of questions, as well as the time it takes to complete the survey. The results of the pilot study showed questions were comprehensible and valid, and therefore, no further edits were made to the survey. The survey was composed of five sections, as follows: (i) socio-demographics (n = 4 questions), (ii) obstetric and menstrual history risk factors of BC (n = 8 questions), (iii) common non-obstetric risk factors of BC (n = 15 questions), (iv) general and signs knowledge about BC (n = 10 and 7 questions, respectively), and (v) sources of knowledge about BC (n = 6 options). 

*Statistical analysis*

The Statistical Package for the Social Sciences (SPSS) software version 22.0.0.0 (SPSS Inc., Chicago, IL, US) was used to analyze the data. Descriptive data were reported as numbers and percentages. For categorical data, two-tailed chi-square test was used. A p value < 0.05 was regarded as statistically significant. For knowledge questions, any correct answer was given a score of one point, whereas any wrong answer was given a score of zero. For the questions pertaining to general knowledge about BC (n = 10 questions), ‘good’ knowledge was defined as ≥6 correct answers. For questions pertaining to signs knowledge about BC, ‘good’ knowledge was defined as ≥4 correct answers. For computing the overall knowledge (that is, general knowledge plus signs knowledge), ‘good’ knowledge was defined as ≥ 9 cumulative correct answers.

## Results

*Socio-demographics*

Table 1 displays the socio-demographical data of the surveyed research participants. Overall, a total of 300 individuals participated in the study. There were 232 students (77.3%) and 68 faculty staff (22.7%). Moreover, the vast majority of research participants were single (76.7%), <25 years of age (71.7%) and had bachelor’s and/or advanced degree (74.7%). 

*Menstrual history and obstetric risk factors of BC*

Table 2 depicts the menstrual history and obstetric risk factors of BC among the surveyed research participants. The vast majority of research participants were still menstruating (96.3%), had menarche after 12 years of age (71.3%) and self-reported regular menstrual cycles (65%). A total of 50 research participants were pregnant at least once in their lives (16.7%). Among those (n=50), the bulk proportion of them was less than 50 years of age when they had their first pregnancy (92%) and reported having between one to three children (84%) at the time of study. Moreover, 46 research participants self-reported breastfeeding their children (92%), however, their self-reported duration of <1 year versus 1-2 years of breastfeeding was comparable (45.5 vs. 54.3%, respectively).

*Non-obstetric risk factors of BC*

Table 3 shows the risk factors of BC among the surveyed research participants. Overall, the research participants favorably demonstrated low risk potential for BC. In a descending order, the most pertinent risk factors for BC included lack of physical exercise (66.3%), positive family history of BC (18%) and obesity (16.7%). Of note, 1.7% and 8.7% of research participants reported occasional smoking and intake of oral contraceptive pills, respectively. 

*General and signs knowledge of BC*

**Figure 1:**
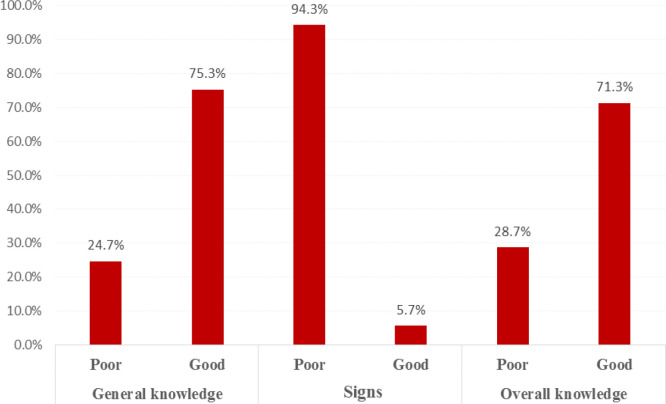
Overall Knowledge about Breast Cancer among Sampled Students and Staff from Najran University, Saudi Arabia

Table 4 exhibits the knowledge level of BC among the surveyed research participants. Overall, research participants demonstrated far higher percentages of correct answers for the general knowledge domain than for the signs knowledge domain. Figure 1 shows the knowledge of BC (general knowledge domain, signs knowledge domain and overall knowledge) among the surveyed research participants. According to pre-defined cutoff criteria, the proportions of surveyed subjects with ‘good’ knowledge in the general and signs knowledge domains were 75.3% and 5.7%, respectively. For all surveyed subjects, the proportion of surveyed subjects with ‘good’ overall knowledge (including general and signs knowledge domains) was 71.3%. 

*Determinants of BC knowledge*

Table 5 displays the socio-demographical determinants of BC knowledge among the surveyed research participants. The statistically significant predictors of ‘good’ overall knowledge were age, social status, educational level, academic status and positive family history of BC (p<0.05).

*Sources of information about BC*

**Figure 2:**
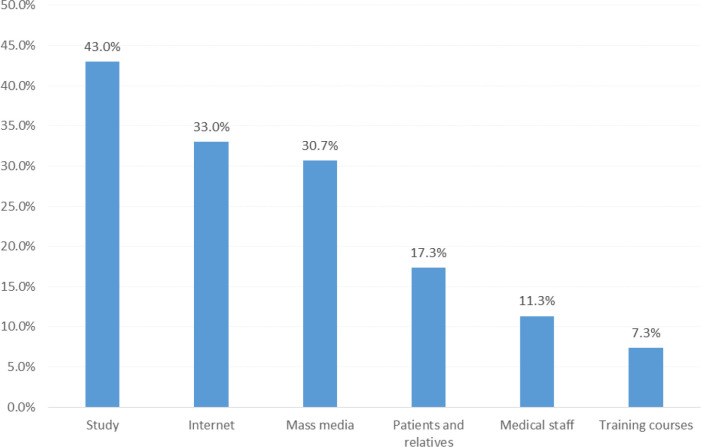
Sources of Knowledge about Breast Cancer among Sampled Students and Staff from Najran University, Saudi Arabia

Figure 2 depicts the sources of information about BC among the surveyed research participants. Apart from the study’s educational awareness campaign (43%), the top two knowledge sources about BC were internet (33%) and mass media (30.7%). Interestingly, BC knowledge provided by healthcare professionals was only 11.3%.

**Table 1:** Personal Data of Sampled Female Students and Staff in Najran University, Saudi Arabia

Personal data		n	%
Age in years	<25 years	215	71.7
	25-39	66	22.0
	40+	19	6.3
Marital status	Single	230	76.7
	Married	62	20.7
	Divorced	8	2.7
Educational level	Secondary/ diploma	76	25.3
	Bachelor	178	59.3
	Master	24	8.0
	Ph.D.	22	7.3
Job	Student	232	77.3
	Staff	68	22.7

**Table 2:** Obstetric and Menstrual History of Sampled Students and Staff from Najran University, Saudi Arabia

Obstetric and menstrual history		n	%
Number of pregnancy (n = 70)	None	20	28.6
	1-3	40	57.1
	4+	10	14.3
Age at first pregnancy (n = 50)	<30 years	46	92.0
	>30 years	4	8.0
Number of children (n = 50)	1-3	42	84.0
	4+	8	16.0
Child was breastfed (n = 50)	Yes	46	92.0
	No	4	8.0
Duration of breast feeding (n = 46)	<1 year	21	45.7
	1-2 years	25	54.3
Age at menarche (n = 300)	<12 years	86	28.7
	>12 years	214	71.3
Regular cycle (n = 300)	Yes	195	65.0
	No	105	35.0
Age of menopause (n = 300)	Still menstruating	289	96.3
	<50 years	8	2.7
	>50 years	3	1.0

**Table 3:** Risk Factors of Breast Cancer among Sampled Students and Staff from Najran University, Saudi Arabia

Breast cancer risk factors		n	%
Family history of breast cancer (n = 300)	Positive	54	18
	Negative	246	82
Used contraceptive pills (n = 300)	Yes	26	8.7
	No	274	91.3
Duration of using pills (n = 26)	<1 year	14	56.0
	>1 year	12	44.0
Used drugs to regulate cycle (n = 300)	Yes	33	11.0
	No	267	89.0
Duration of used drug (n = 33)	<1 year	21	63.6
	1 year	6	18.2
	2 years	3	9.1
	3 years	3	9.1
Received hormonal therapy (n = 300)	Yes	25	8.3
	No	275	91.7
Had benign breast mass (n = 300)	Yes	13	4.3
	No	287	95.7
Regular exercise (n = 300)	Yes	101	33.7
	No	199	66.3
Had depression (n = 300)	Yes	36	12.0
	No	264	88.0
Psychological stress (n = 300)	Yes	114	38.0
	No	186	62.0
Undergone breast plastic surgery (n = 300)	Yes	1	0.3
	No	299	99.7
Had radiotherapy (n = 300)	Yes	17	5.7
	No	283	94.3
Smoking (n = 300)	Yes	5	1.7
	No	295	98.3
Body mass index (kg/m^2^) (n = 300)	Normal	201	67.0
	Overweight	49	16.3
	Obese	50	16.7

**Table 4:** Knowledge about Cancer among Sampled Students and Staff from Najran University, Saudi Arabia

Knowledge domain	Items	Correct answer n (%)
General Knowledge	Smoking causes BC	273 (91.0)
	Heard about BC	264 (88.0)
	BC can be discovered early	222 (74.0)
	Early detection of BC helps in treatment	221 (73.7)
	BC is common in the community	184 (61.3)
	Know about breast self-examination	225 (75.0)
	Know about benign breast masses	223 (74.3)
	Attended breast self-examination educational sessions	231 (77.0)
	Attended breast cancer self-examination educational sessions	239 (79.7)
	Know about mammogram	243 (81.0)
Signs Knowledge	Nipple discharge	35 (11.7)
	Breast mass	29 (9.7)
	Skin changes	34 (11.3)
	Change in nipple shape and size	27 (9.0)
	Change in breast size	31 (10.3)
	Change in breast height	30 (10.0)
	Axillary mass	17 (5.7)

**Table 5:** Determinants of Breast Cancer Awareness among Sampled Students and Staff in Najran University, Saudi Arabia

Factors		Overall knowledge				*p*-value
		Poor		Good		
		n	%	n	%	
Age in years	<25 years	76	35.3	139	64.7	0.0001*
	25-39	10	15.2	56	84.8	
	40+	0	0	19	100.0	
Marital status	Single	78	33.9	152	66.1	0.0003*
	Married	5	8.1	57	91.9	
	Divorced	3	37.5	5	62.5	
Educational level	Secondary/ diploma	45	59.2	31	40.8	<0.0000*
	Bachelor’s	39	21.9	139	78.1	
	Master’s	2	8.3	22	91.7	
	Ph.D.	0	0.0	22	100.0	
Job	Student	85	36.6	147	63.4	<0.0000*
	Staff	1	1.5	67	98.5	
Number of pregnancy	None	5	25	15	75.0	0.0627
	1-3	3	7.5	37	92.5	
	4+	0	0.0	10	100.0	
Number of children	1-3	3	7.1	39	92.9	0.4356
	4+	0	0.0	8	100.0	
Family history of breast cancer	Positive	9	16.7	45	83.3	0.0313*
	Negative	77	31.3	169	68.7	
Had benign breast mass	Yes	4	30.8	9	69.2	0.8639
	No	82	28.6	205	71.4	

* Statistical significance as *p*-value <0.05, two-tailed Chi-square test

## Discussion

Earlier studies from Saudi Arabia have specifically investigated the women’s awareness level of BC risk factors (Amin et al., 2009; Radi, 2013; Alrashidi et al., 2017; Al-Zalabani et al., 2018; Binhussien and Ghoraba, 2018; Alshahrani et al., 2019b). Collectively, these studies showed dissimilar results. To elaborate, the awareness level of BC risk factors was satisfactory among women from Riyadh (central region), whereas the awareness level of BC risk factors was dramatically low among women from Hail (northern region), Al-Ahsa (eastern region), Asir (southern region), Jeddah (western region) and Madinah (western region). These findings raise the plausible concern as whether awareness level varies among different regions of Saudi Arabia; this matter warrants further investigation and mandates more targeted awareness campaigns in such regions. 

The only published study from Najran did not examine the actual risk factors of BC among the surveyed women attending primary healthcare centers (Alshahrani et al., 2019a). Thus, our study sought to explore the most common risk factors of BC among a sampled female population (students and faculty) from Najran University. Our data disclosed that the most prevalent reproductive risk factors of BC were early menarche before 12 years of age (29.7%) and nulliparity (83.8%). Early age at the time of menarche (less than 12-13 years) has been demonstrated to yield a higher risk of estrogen receptor-positive (ER+) and progesterone receptor-positive (PR+) BC (Ritte et al., 2013). Moreover, several reports have established that nulliparity potentiates a higher risk of BC (Rosner et al., 1994). The high rate of nulliparity (83.8%) should be interpreted with caution, as the vast majority of surveyed participants were single (76.7%). Nevertheless, appropriate awareness of the modifiable and non-modifiable risk factors of BC is very critical to stratify high-risk women who have higher propensity toward development of BC.

With regard to non-reproductive risk factors of BC, our findings showed that a large proportion of females reported a lack of physical exercise (66.3%). A meta-analysis of prospective studies showed that regular physical activity seems to be a protective factor against development of BC in the absence of hormonal replacement therapy (HRT) use (Pizot et al., 2016). From a biological perspective, physical activity may lessen the hazard of BC pathogenesis through reduction of important BC-promoting hormones-for example, insulin, insulin-like growth factor type 1 (IGF-1) and IGF-1 binding protein type 3 (IGFBP-3) serum blood levels (Irwin et al., 2019). The substantial lack of physical activity in this cohort mandates devising curricular, extracurricular and institutional measures geared toward facilitating and encouraging physical exercise.

Our data showed that while the surveyed research subjects had ‘good’ general knowledge about BC, they demonstrated ‘poor’ signs knowledge about BC. With regard to general knowledge about BC, the surveyed females claimed familiarity with BSE as well as attending educational sessions about BSE. However, these claims could not be verified in our study, and whether the surveyed women are truly able to conduct BSE ‘properly’ remains unknown. An earlier report disappointingly showed that only a very negligible fraction of women (less than 2%) out of 157 women surveyed in Jeddah (western region) were able to demonstrate proper conduct of BSE (Kashgari and Ibrahim, 1996). With regard to ‘poor’ signs knowledge about BC, our findings were no different and mirrored the outcomes of earlier studies (Radi, 2013; Al Otaibi et al., 2017; Alrashidi et al., 2017; Binhussien and Ghoraba, 2018; Alshahrani et al., 2019b). These results collectively trigger a pressing need to exert serious efforts wheeled toward enriching the knowledge of women about the warning early signs of BC. In fact, these awareness campaigns should be extended further and include the males (husbands in particular) as they are equally paramount partners in promoting awareness of BC among their wives, daughters and significant females (Al-Musa et al., 2019).

A number of studies from Saudi Arabia showed that the overall awareness level of BC varied substantially according to diverse socio-demographical parameters, such as age, gender, marital status, occupation, monthly income, educational level and family history (Amin et al., 2009; Radi, 2013; Al Otaibi et al., 2017; Alshahrani et al., 2019b). In our study from Najran of surveyed female students and faculty, the predictors of ‘good’ overall knowledge included age, marital status, educational level and family history (p = 0.001). A study from Najran by Alshahrani and partners surveyed female patients attending primary healthcare centers (Alshahrani et al., 2019a); only educational level and family history were in agreement with our predictors of ‘good’ overall knowledge. The differences in populations (that is, primary healthcare centers versus university setting) may have played an attributable role to such differences in the results.

Our findings depicted that the top two sources of knowledge about BC were internet and mass media. These findings were in agreement with a previous study from Najran (Alshahrani et al., 2019a), as well as other studies from different regions in Saudi Arabia (Binhussien and Ghoraba, 2018; Alshahrani et al., 2019b). It is very worrisome that the healthcare professionals were not a major source of knowledge about BC among our sampled cohort of research participants, and this alarming concern has been echoed in other studies, too (Al Otaibi et al., 2017; Binhussien and Ghoraba, 2018; Alshahrani et al., 2019a; Alshahrani et al., 2019b). In spite of the availability of free facilities for BC screening in Saudi Arabia (including CBE and mammogram), the participation rate of women is extremely low with almost no takers (El Bcheraoui et al., 2015). This low participation rate may be largely ascribed to the dearth of information about the process and advantages of seeking such screening services for BC. This matter is further complicated by the lack of a straightforward (transparent and clear) description of a nationwide screening program for BC (Gosabi, 2019). Despite several governmental and non-governmental awareness campaigns about BC take place across the country, such campaigns lack fundamental qualities. For example, focus group discussions with women from Makkah depicted that such awareness campaigns are done in specific months (that is, seasonal campaigns only during the month of October) and not throughout the year to accommodate the convenience of women (Nageeti et al., 2017). Moreover, shopping malls are not the most suitable spots to conduct awareness campaigns (Nageeti et al., 2017). It is time to upgrade the quantity and quality of BC awareness campaigns nationally, so that these orchestrated missions will auspiciously yield tangible benefits. Recently, Alanzi et al., (2018) explored the utility of SnapChat mobile social networking application in prompting awareness of BC among the Saudi population, and the results were very favorable.

Our study has several strengths. First, it is the second ever study conducted from Najran region about the awareness level of BC among female students and faculty staff, which is in contrast to the population of women attending primary healthcare centers that was reported by Alshahrani et al., (2019a). Second, our study included a relatively large sample size of 300 participants. Third, our study explored obstetric and non-obstetric risk factors of BC, which was not previously explored by an earlier study (Alshahrani et al., 2019a). Overall, our study enriches the literature on the topic of BC awareness and contributes new knowledge from an underrepresented region in Saudi Arabia. Nevertheless, our study has limitations. Such limitations include the self-reported responses of the participants, which could not be verified and were liable to over- or under-estimation. Additional limitation include the lack of assessing the perceived barriers toward BC screening, such as BSE, CBE and mammography, which will be addressed in a forthcoming study. 

In conclusion, we surveyed a sampled population of female students and faculty (n = 300) from Najran University, Najran, Saudi Arabia. Our study showed that early menarche before 12 years of age and nulliparity were the most pertinent obstetric risk factors of BC among the surveyed research subjects. On the other hand, lack of physical activity was the most substantial non-obstetric risk factor of BC. The surveyed research subjects demonstrated ‘poor’ knowledge about the warning early signs of BC. The predictors of ‘good’ overall knowledge included age, marital status, educational level and family history. Apart from the campaigns’ educational sessions, the top sources of knowledge about BC were internet and mass media, whereas the lowest ones were healthcare professional and training workshops. Finally, we call for more rigorous and well-crafted educational campaigns geared toward improving the awareness level of BC among women in Najran region. 
